# The validity of teledentistry examinations during the COVID‐19 pandemic in Sydney, Australia

**DOI:** 10.1111/adj.13053

**Published:** 2024-12-16

**Authors:** T Mahony, A George, SG Kezhekkekara, A Villarosa, C Friel, A Yaacoub

**Affiliations:** ^1^ Nepean Centre for Oral Health Nepean Blue Mountains Local Health District Kingswood New South Wales Australia; ^2^ Australian Centre for Integration of Oral Health (ACIOH), School of Nursing & Midwifery Western Sydney University Liverpool New South Wales Australia; ^3^ Ingham Institute for Applied Medical Research Liverpool New South Wales Australia; ^4^ School of Dentistry, Faculty of Medicine and Health The University of Sydney Sydney New South Wales Australia; ^5^ School of Nursing University of Wollongong Wollongong New South Wales Australia; ^6^ National Centre of Epidemiology and Population Health Australian National University Canberra Australian Capital Territory Australia

**Keywords:** Telehealth, teledentistry, consultations, oral health, model of care, dental treatment, corona virus, COVID‐19, SARS‐CoV2, diagnoses, sensitivity, specificity

## Abstract

**Background:**

During the COVID‐19 pandemic, non‐urgent dental treatments were deferred. To identify and prioritize urgent dental patients, teledentistry was implemented across NSW public dental services. This study aimed to establish the validity of teledentistry examinations to provide a clinical diagnosis compared to face‐to‐face, clinical examination.

**Methods:**

A retrospective review using convenience sampling was used to source clinical data from a public dental service for two periods in 2020 and 2021. Researchers compared the diagnoses identified from teledentistry consultations with follow‐up face‐to‐face consultations, diagnoses were grouped into 14 sub‐categories that broadly covered common oral health conditions and pathologies. Sensitivity, specificity, NPV and PPV were calculated.

**Results:**

The study included 1965 patients who underwent teledentistry followed by face‐to‐face consultations, with a mean age of 47.79 ± 21.92 years. Teledentistry showed high specificity ranging from 74.08% to 99.95% while the sensitivity ranged from 18.75% to 100%. The highest specificity (99.90%) was noted for diagnosing orthodontic concerns. The lowest specificity was observed for irreversible pulpal diseases at 74.08%.

**Conclusion:**

Teledentistry has a role in public dental services, diagnosing various dental conditions and identifying patient acuity. It can provide patients with oral health information/advice and ensures effective use of emergency appointments within public dental services.

**Abbreviations and acronyms**
AAE: American Association of Endodontists
ACIOH: Australian Centre for the Integration of Oral Health
ADA: Australian Dental Association
AHPPC: Australian Health Protection Principle Council
ISO: International Organization for Standardization
MoH: Ministry of Health
NPV: negative predictive value
PPV: positive predictive values
ROC: receiver operating characteristic

**Clinical Relevance**

Dental caries and periodontal disease pose a significant global health burden, affecting 3.5 billion people. The COVID‐19 pandemic disrupted access to dental services in Australia, necessitating a shift to teledentistry. Previous evidence suggests teledentistry's potential for remote diagnosis and patient management, especially in emergency triaging. However, there is little known about the validity of teledentistry for diagnosing oral conditions compared to face‐to‐face clinical examinations within the Australian context. This research study confirms its reliability, whilst providing a robust alternative for initial consultations and managing dental emergencies when face‐to‐face dental consultations are not readily available, for example, pandemic‐related restrictions. However, the authors propose that further research is required to support the integration of teledentistry in clinical practice.

## INTRODUCTION

Dental caries and periodontal disease have been considered a significant global health burden with oral disease thought to be affecting nearly 3.5 billion people globally. Many health consumers are reliant upon dental services to prevent or reduce the likelihood of the sequalae of oral disease.^1^, ^2^ However, access to dental services was significantly impacted and reduced following the outbreak of the Novel Coronavirus (COVID‐19). COVID‐19 was first detected in December 2019 and identified to be a highly communicable disease spread predominantly via droplet, fomite and contact transmission.^3^ By February 2020, COVID‐19 was declared a global pandemic. As a profession reliant on close contact with an individual's nasopharyngeal region, the primary area for COVID‐19 colonization, the dental care professionals were faced with significant challenges due to the risk they and their consumers faced, when undertaking or receiving dental treatment.^4^

Leading dental organizations both nationally and internationally responded by offering dental practitioners needed direction and recommendations. In most countries, dental care services were limited to urgent cases during the COVID‐19 pandemic. The implementation of strict infection control measures was recommended to prevent the spread within healthcare settings. In Australia, the Australian Dental Association (ADA) and Australian Health Protection Principle Council (AHPPC) provided key guidance to dental professionals regarding dental treatment.^5^ Due to the rapid transmissibility of COVID‐19, the issued guidance advised deferring dental treatments based on urgency.^6^ The guideline provided escalating levels of restrictions on dental services, with the most severe level resulting in no routine dental treatment to be provided, and all patients with acute dental concerns being directed to emergency care centres. Research into the impact COVID‐19 has had on the delivery of dental services in Australia has shown a 52% decrease in the number of dental services provided during the first wave, and this pattern was replicated during the second wave in 2021.^7^ This widespread disruption created a critical need for alternative models of care that could safely deliver dental services without requiring face‐to‐face consultations.

Telemedicine is a concept that has been utilized and researched prior to the COVID‐19 pandemic and has been noted to produce benefits, both economic and clinically, for both the health system and consumer.^8^, ^9^ As a branch of telemedicine, teledentistry was first implemented in 1994 as a project for increasing access to dental services in the US army.^10^ Teledentistry has been utilized via a number of modalities including store and forward consultations for referrals/triage patients and real‐time consultations between GPs and the dental team.^11^ Since then, the dental profession has experienced a rapid uptake in the use of communication and information‐based technologies for engaging in oral health promotion and improving consumer experience. Engagement and widespread adoption of this technology was further accelerated during the pandemic.^5^, ^12^ In response to the implemented guidelines and dynamic pandemic environment, dental services engaged in teledentistry allowing the triaging of consumer oral health concerns for prioritization of dental emergencies and scheduling of non‐urgent cases.

From the consumer point of view, studies investigating the implementation and acceptability of teledentistry during the COVID‐19 pandemic have shown that patients were highly satisfied with the management of their dental concerns via teledentistry and were generally accepting of the service.^12^, ^13^ Further, in a review of the application of teledentistry during the COVID‐19 pandemic, the authors discussed the benefits of teledentistry in being able to provide specialist dental consultation, rapid management or early intervention of preliminary dental emergencies and reducing the need for follow‐up consultations.^5^, ^14^ Teledentistry was also noted to be a viable option for providing early intervention counselling or consultation among a broad range of population groups from paediatrics to aged care and across a variety of specializations including endodontics, orthodontics, periodontics and prosthodontics.^3^, ^14^, ^15^, ^16^, ^17^

As a response to the COVID‐19 pandemic waves in Australia, teledentistry consultations in the state of New South Wales were used on a broad scale following the 2020 Ministry of Health (MoH) teledentistry guidelines and the ADA's guidelines on performing routine dental care during the first and second COVID‐19 waves across NSW, Australia.^18^ Under these circumstances, while some services continued to be performed in public dental clinics, many patients were managed with the aid of teledentistry. This eliminated unnecessary or preventable patients' presentation to the dental clinic, therefore minimizing risk to patients and staff during a period of high community transmission of COVID‐19.

Overall, reviews exploring the implementation and acceptability of teledentistry indicate that this model has a place in dentistry; however, there is limited evidence to support the validity of teledentistry consultation versus diagnosis from the standard face‐to‐face clinic appointment (Alabdullah and Jampani^10^, ^16^). In a review published in 2018, the authors concluded that teledentistry can be comparable to face‐to‐face for oral screening and is possible for identification and diagnosis of oral disease and referral.^16^ Within the Australia context, there is limited evidence exploring the validity of teledentistry to provide a clinical diagnosis, when compared to a direct clinical examination. The objectives of this study were:

1. To evaluate the validity of teledentistry in diagnosing oral conditions when compared to a traditional face‐to‐face clinical examination within the Australian context.
2. To determine whether teledentistry consultations followed by face‐to‐face examinations within a 7‐day period, provided comparable diagnostic accuracy.
3. To evaluate the effectiveness of teledentistry in triaging dental emergencies and managing non‐urgent cases during periods of restricted access to dental care, such as during the COVID‐19 pandemic.

It was hypothesised that teledentistry would demonstrate a high degree of diagnostic accuracy in identifying oral health conditions and would be comparable to diagnoses made through face‐to‐face clinical examinations. Teledentistry was also thought to be able to effectively triage dental emergencies, therefore reducing the need for immediate face‐to‐face consultations during the COVID‐19 pandemic, without compromising patient safety and patient outcomes.

This study is part of a larger project, which explored the current perceptions of teledentistry as a model of care by dental clinicians, and found that, although teledentistry was a novel concept for most dental clinicians, it was well accepted by participants of the study (Mahony *et al.* ^19^).

## MATERIALS AND METHODS

### Study design

This retrospective clinical study was conducted using data from a routine clinical audit across a single public health organization, with four dental clinics in the Western Sydney region. All four clinics were included into the study, as patients were directed to attend their local public dental clinic following the teledentistry consultation. These clinics were selected as they were providing services during the COVID‐19 lockdown period.

### Study setting

Teledentistry services at the study site were introduced following review of the 2020 Ministry of Health (MoH) teledentistry guidelines and the ADA's guidelines on performing routine dental care during the 2020 and 2021 COVID‐19 pandemic. The guideline provided five levels of restrictions on dental services as summarized in Table 1.^20^ Under these circumstances, while some services continued to be performed in the public dental clinics, many patients' dental complaints were managed with the aid of teledentistry. During the peak of dental restrictions, teledentistry consultations were utilized at large scale and assisted oral health staff to clinically triage patients, determine the acuity of their dental complaint, their COVID‐19 risk status and provide an appropriate management pathway. Therefore, it became important to provide dental clinicians with the appropriate training to assist with the completion of these teledentistry consultations, as well as to ensure a systematic and standardized approach to patient care.

**Table 1:** ADA Dental Service Restrictions in COVID‐19

Category	Restrictions towards dental treatment/service
Level 5	No routine dental treatment provided. All patients with acute dental concerns to be directed to emergency care centres.
Level 4	Defer all dental treatments for patients requiring non‐urgent care.
Level 3	Defer all routine recall examinations and dental treatments for patients not fitting the risk categories (pain, swelling, bleeding and difficulty swallowing) Only dental treatments that do not generate aerosols, or where aerosol‐generating treatment is limited to the management of patients with acute dental pain, significantly damaged upper front teeth, soft tissue pathology or those at high risk of rapid progression of dental disease may be completed.
Level 2	Defer all treatments that are likely to generate aerosols which may include the use of high‐speed handpiece without rubber dam, etc. Provision of dental treatments that are unlikely to generate aerosols or where aerosols generated have the presence of minimal saliva/blood due to the use of rubber dam.
Level 1	All dental treatments using standard precautions for people who do not meet epidemiological or clinical risk factors for COVID‐19 infection transmission. Defer non‐urgent treatment for people who DO meet epidemiological or clinical symptom criteria for COVID‐19 risk.
No restrictions	All dental services can be performed. No restrictions apply.

### Teledentistry consultations

During the COVID‐19 pandemic restrictions on dental services, patients (or parent/guardian of a patient) contacted the oral health service via a call‐centre telephone number, where non‐clinical staff used a scripted questionnaire to identify the priority and eligibility of the patient.^21^, ^22^ Patients with urgent or acute problems were recommended a teledentistry consultation and were subsequently contacted via telephone call by the consulting clinician to ascertain the patients chief complaint and symptoms. Images were often requested from patients to assist with the interpretation of symptoms or the diagnosis; however, this was not always possible and often photographs were not useful. All images were transmitted to corporate Local Health District mobile devices only and no personal devices were utilized. These images were then transferred from corporate devices to the service imaging managing system and saved into the patient's unique electronic record.

During the teledentistry consultation, the clinician utilized their clinical experience to evaluate the urgency of the patient's condition based on reported symptoms and a photograph if provided. Established protocols for triaging dental emergencies were utilized to determine which conditions required a follow‐up, face‐to‐face dental visit, as well as to determine the potential risks associated with the patient's conditions, such as pain severity, the likelihood of complications if left untreated and the patients overall health status. This process was established to justify face‐to‐face dental visits due to the imposed restrictions on face‐to‐face dental visits and the need to avoid unnecessarily travelling within the community. If the teledentistry diagnosis ascertained an urgent or acute condition or if it was unclear, the clinician would then schedule the patient to attend a face‐to‐face consultation and treatment appointment at one of the four local public dental clinics. If the teledentistry consultation concluded that no immediate or urgent care was required, the patient was placed on a suitable waiting list for future care once restrictions lifted. The duration of the teledentistry consultations varied, typically ranging from 15 to 20 min.

### Sampling strategy and eligibility

Convenience sampling was used to source data from records of patients who had a teledentistry consultation between 27 March 2020 and 1 September 2020 and between July 2021 and September 2021 across the study sites.

**Fig. 1:**
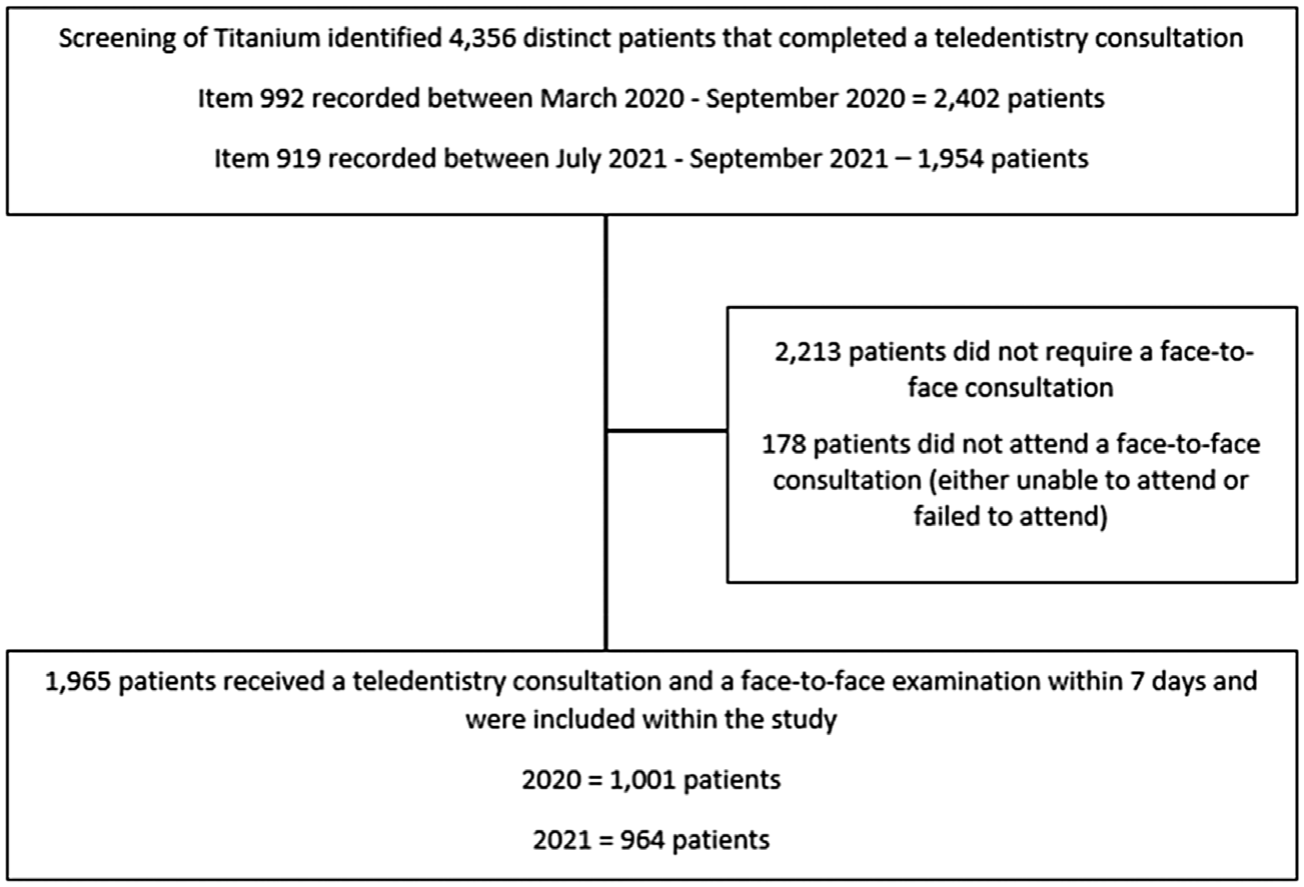
CONSORT flow diagram.

As seen in Fig. 1, patient records were included in the clinical audit if the patient had completed a teledentistry consultation followed up with a face‐to‐face, direct clinical examination within 7 days, which was determined through the review of the electronic dental records ‘Titanium’. Titanium is a comprehensive electronic oral health record system used within NSW public oral health services to document and manage patient information. It allows dental professionals to schedule appointments, record clinical notes, document and create treatment plans, and other essential data, ensuring accurate and efficient patient care. A report of the dental treatment item numbers which are used as the standard coding system of dental treatment in NSW public dental services identified patients who received item numbers 992 ‘Teledentistry live remote/provider‐end’ or 919 ‘the provision of teledentistry consultation’. The item number 992 was used for teledentistry consultations completed between March 2020 and September 2020, whereas the item number 919 was used between July 2021 and September 2021.

Records were only selected for the study if the patient met the following teledentistry selection criteria:

- Adult and child individuals who were eligible for public dental care at the study site according to the Ministry of Health eligibility for public dental service policy document.^22^
- Patients from the study site who required emergency or acute dental care during Level 2 and 3 dental restrictions (between the dates of 25 March 2020 and 29 September 2020 and between 28 July 2021 and 30 September 2021) and identified as a Code 1, Code 3A or Code 3B by the Ministry of Health ‘priority oral health program’ policy document^22^ and coded accordingly in the electronic oral health record ‘Titanium’.
- Participants (patients and parent/guardians of a patient) had completed a teledentistry consultation in the above‐mentioned time period.
- Patients who had attended a follow up face‐to‐face clinical examination with a dental clinician within 7 days of the teledentistry consultation.

### Ethical considerations

Ethics approval for this study was obtained from the Human Research Ethics Committee of Nepean Blue Mountains Local Health District (HREC Ref No. 2021/ETH00394).

### Data collection

Based on meeting the eligibility criteria, a search was conducted for item number 919 ‘the provision of teledentistry consultation’ and 992 ‘Teledentistry live remote/provider‐end’ within the electronic patient database (titanium) during the study time period.^23^ Clinicians who were involved in teledentistry consults followed a standardized electronic template in the dental record for gathering clinical information and recording their diagnosis and treatment. This template included a list of clinical questions relating to presenting complaint, onset, duration and severity of symptoms (and photographs sent by the patient if possible) to complete the teledentistry consultation. A provisional diagnosis, outcome and actions of the teledentistry consultation were then recorded by the clinician. The researchers extracted the retrospective data from the patient's electronic health record on Titanium and systematically entered it into an Excel spreadsheet for organization and analysis.

Researchers used the information in the dental records to determine both the provisional diagnosis identified during the teledentistry consultation, as well as the clinical diagnosis identified during the subsequent face‐to‐face consultation appointment.

To ensure accurate and consistent diagnoses within a teledentistry setting, the researchers aimed to use standardized diagnostic terminology to cover the spectrum of dental conditions and pathologies. Based on the current literature, there is no consensus on the entire spectrum of dental conditions and pathologies including specific terminology to be used or how it should be implemented.^24^ Standardized dental diagnostic terminology varies across different countries, influenced by local practices, regulatory frameworks and the integration of international standards. Countries may adopt different versions or interpretations of these standards based on local needs and practices. This study was conducted in New South Wales, Australia, which is predominately aligned with the International Organization for Standardization (ISO)/TC 106. ISO TC 106 is specifically focused on dental standards and includes inputs from numerous countries. Similarly, the American Association of Endodontists (AAE) diagnostic terminology plays a significant role in standardizing pulpal‐related conditions and endodontic diagnosis^25^ which has become widely used in both North America and Australia. When conducting teledentistry consultations, the AAE diagnostic criteria for pulpal conditions were applied, as this standardized approach facilitated the comparison of teledentistry diagnoses with those obtained through face‐to‐face examinations.

Unfortunately, there is a notable absence of defined criteria for diagnosing other dental conditions. Therefore, to address this issue, researchers applied distinct groups of dental conditions/pathologies and their associated diagnoses (see Table 2). These groups include terms from the AAE classification, supplemented by other diagnoses determined by the researchers themselves (blinded/redacted). The researchers were also guided by the diagnostic grouping applied in recently published, similar studies.^10^, ^16^ The inter‐rater reliability was assessed using ICC statistics to ensure that the researchers were consistent in their ability to interpret the data. The ICC obtained using two‐way mixed effects model was 0.835 (95% CI: 0.642–0.941, *P* < 0.001) indicative of good reliability.^26^

**Table 2:** Dental conditions and diagnosis categories ranked by the most common diagnoses occurring in the direct, face‐to‐face examination

Diagnoses Categories	Occurrence (n)	Prevalence (%)
Irreversible pulpitis, pulpal necrosis and apical periodontitis	1012	51.50
Reversible pulpitis and hypersensitivity	379	19.29
Periodontal diseases	203	10.33
Crown fracture or defective restoration with no associated pulpitis	132	6.72
Pericoronitis and wisdom teeth conditions	101	5.14
Diagnosis of non‐dental origin and unknown diagnosis	64	3.26
Lost, ill‐fitting or broken dentures or dental prosthetics	52	2.65
Dental trauma	26	1.32
Soft tissue lesions and ulcers	23	1.17
TMD/TMJ conditions	21	1.07
Postoperative complications including dry socket	14	0.71
Mobile and exfoliating deciduous teeth	13	0.66
Orthodontic concerns including orthodontic appliances	12	0.61
Total	1965	100

Demographic information including gender, age, indigenous status and local government area was also collected.

### Statistical analysis

All analyses were undertaken in STATA statistical software (Version 17.0; StataCorp). Descriptive statistics were computed to summarize the collected data. To assist with analysis, diagnoses were grouped into categories that correspond to the general dental problem that was diagnosed (Table 1). Sensitivity, specificity, negative predictive value (NPV) and positive predictive values (PPV) were calculated. Specificity is the ability to correctly identify those without a dental problem and sensitivity is the ability to correctly identify those with a dental problem. While PPV and NPV report the extent to which tele‐dentistry can avoid reporting false positive and false negative cases of dental problems. Receiver operating characteristic (ROC) analyses were conducted to determine the diagnostic performance of teledentistry. *P* value of <0.05 was taken to indicate statistical significance. The accuracy of teledentistry diagnoses was benchmarked against the outcomes of the direct face‐to‐face examination, which was used as the Gold Standard in this study. For example, if the patient's face‐to‐face diagnosis was irreversible pulpitis, the researchers considered this to be the gold standard diagnosis. Therefore, if the teledentistry diagnosis was also irreversible pulpitis, it was considered to match the gold standard diagnosis.

The authors analysed the results collectively and did not look for any differences among the four individual centres, as the teledentistry clinicians consulted across the four clinics within the same local health district, and patients were offered face‐to‐face consultation appointments at their closest located clinic to prevent unnecessary travel within the community. The teledentistry clinicians often consulted for patients who would be attending one of the other four clinics from where they were consulting.

## RESULTS

### Demographics

Based on the direct examinations, the most reported diagnostic category was irreversible pulpitis, pulpal necrosis and apical periodontitis (51.50%) and the least common category was orthodontic concerns including problems with orthodontic appliances (0.61%). The prevalence percentage of all the different diagnosis categories is listed in Table 2. In total 1965 patients had completed both a teledentistry consultation and attended a dental appointment, where a face‐to‐face examination was completed and were therefore included in this study. The mean age of the patients was 47.79 ± 21.92 years and 75% were in the age range of 15–70 years. The records included 60% females and about 40% of males. Around 7% of the records were of indigenous Australians. Table 3 lists the sociodemographic characteristics of the patients in detail.

**Table 3:** Demographic characteristics of patients

Demographics	n (%)
Year of consult	
2020	1001 (50.94%)
2021	964 (49.06%)
Gender	
Male	785 (39.95%)
Female	1180 (60.05%)
Age^a^ (mean ± SD)	44.79 (SD 21.92)
<15 years old	180 (9.18%)
15–70 years old	1477 (75.32%)
>70 years old	304 (15.50%)
Photographs obtained	
Yes	1601 (81.48%)
No	364 (18.52%)

^a^ Three missing values, n = total no: of patients.

### Validity of teledentistry as compared to face‐to‐face clinical diagnoses

The teledentistry diagnosis was assessed against the gold standard diagnosis obtained from the direct clinical examination. The analyses found teledentistry to have specificity ranging from 74.08% to 99.95% and sensitivity ranging from 18.75% to 100%. Table 4 lists sensitivity and specificity of teledentistry for the various diagnoses in detail. More specifically, highest specificity (99.90%) and sensitivity (100%) were noted for diagnosing orthodontic concerns including problems with orthodontic appliances. Here the PPV was 85.71% and the NPV was at 100%. Furthermore, in ROC analyses the area under the curve for diagnosing orthodontic issues was 0.93 (95% CI: 0.916–0.940) (Table 5). Similarly, the lowest specificity was observed for irreversible pulpitis, pulpal necrosis and apical periodontitis at 74.08% while its sensitivity was 84.29%. The PPV and NPV values were 77.55% and 81.62%, respectively. It is important to note that this diagnostic grouping was the most relevant in the sample of patients with dental complaints, representing 51.5% of the total study cohort.

**Table 4:** Sensitivity, specificity, NPV and PPV of teledentistry for various diagnoses N = 1965

Diagnosis	Sensitivity (%)	Specificity (%)	PPV (%)	NPV (%)
Irreversible pulpitis, pulpal necrosis and apical periodontitis	84.29	74.08	77.55	81.62
Reversible pulpitis and hypersensitivity	58.84	90.23	58.99	90.17
Periodontal diseases	52.22	97.22	68.39	94.64
Crown fracture or defective restoration with no associated pulpitis	49.24	98.20	66.33	96.41
Pericoronitis and wisdom teeth conditions	86.14	98.55	76.32	99.24
Diagnosis of non‐dental origin and unknown diagnosis	0.1875	99.95	60.00	97.33
Lost, ill‐fitting or broken dentures or dental prosthetics	82.69	99.58	84.31	99.53
Dental trauma	88.46	99.54	71.88	99.84
Soft tissue lesions and ulcers	73.91	98.66	39.53	99.69
TMD/TMJ conditions	47.62	99.79	71.43	99.44
Postoperative complications including dry socket	57.14	99.79	66.67	99.69
Mobile and exfoliating deciduous teeth	76.92	99.90	83.33	99.85
Orthodontic concerns including orthodontic appliances	100	99.90	85.71	100

**Table 5:** ROC area for each diagnosis

Diagnosis	ROC area (95% CI)
Irreversible pulpitis, pulpal necrosis and apical periodontitis	0.792 (0.774–0.810)
Reversible pulpitis and hypersensitivity	0.745 (0.719–0.771)
Periodontal diseases	0.747 (0.713–0.782)
Crown fracture or defective restoration with no associated pulpitis	0.737 (0.694–0.780)
Pericoronitis and wisdom teeth conditions	0.924 (0.886–0.957)
Diagnosis of non‐dental origin and unknown diagnosis	0.592 (0.543–0.640)
Lost, ill‐fitting or broken dentures or dental prosthetics	0.911 (0.859–0.963)
Dental trauma	0.940 (0.927–0.950)
Soft tissue lesions and ulcers	0.863 (0.771–0.955)
TMD/TMJ conditions	0.737 (0.628–0.847)
Postoperative complications including dry socket	0.785 (0.650–0.919)
Mobile and exfoliating deciduous teeth	0.884 (0.869–0.898)
Orthodontic concerns including orthodontic appliances	0.929 (0.916–0.940)

Table 5 lists the ROC area obtained for each diagnosis in detail. Overall, the ROC analyses showed good to excellent values. Higher ROC area was observed for the diagnosis dental trauma (0.94, 95%Cl: 0.927–0.950) indicating excellent discriminative ability of teledentistry. The lowest was observed for diagnosis of non‐dental origin and unknown diagnosis (0.59, 95%Cl: 0.543–0.640). When comparing by age, area under the ROC curve for crown fracture or defective restoration with no associated pulpitis was significantly higher (chi square = 8.55, 1 df, *P* = 0.004) among those ≥15 years of age (0.76, 95%Cl: 0.71–0.80) compared to those <15 years (0.58, 95%Cl:0.467–0.687). Furthermore, when comparing by photographs obtained for diagnosis, area under the ROC curve for pericoronitis and wisdom teeth conditions was significantly higher (chi‐square = 15.43, 1 df, *P* < 0.001) for those with photographs (0.99, 95% CI: 0.984–0.998) compared to those without photographs (0.91, 95% CI: 0.868–0.949).

Furthermore, Table 6 shows the most mistaken diagnosis by teledentistry in each of the diagnosis categories. All cases of orthodontic concerns were correctly diagnosed by teledentistry. However, for all the other categories of diagnoses, teledentistry predominantly misdiagnosed them as irreversible pulpitis.

**Table 6:** Misdiagnosed by teledentistry consultation (%)

Face to face diagnosis	Mostly misdiagnosed by tele dentistry as (%)
Irreversible pulpitis, pulpal necrosis and apical periodontitis	Reversible pulpitis and hypersensitivity (8%)
Reversible pulpitis and hypersensitivity	Irreversible pulpitis, pulpal necrosis and apical periodontitis (34%)
Periodontal diseases	Irreversible pulpitis, pulpal necrosis and apical periodontitis (34%)
Crown fracture or defective restoration with no associated pulpitis	Reversible pulpitis and hypersensitivity (29%)
Pericoronitis and wisdom teeth conditions	Irreversible pulpitis, pulpal necrosis and apical periodontitis (13%)
Diagnosis of non‐dental origin and unknown diagnosis	Irreversible pulpitis, pulpal necrosis and apical periodontitis (44%)
Lost, ill‐fitting or broken dentures or dental prosthetics	Irreversible pulpitis, pulpal necrosis and apical periodontitis (12%)
Dental trauma	Crown fracture & Reversible pulpitis and hypersensitivity (both at 8%)
Soft tissue lesions and ulcers	Crown fracture, Irreversible pulpitis, pulpal necrosis and apical periodontitis, Lost, ill‐fitting or broken dentures or dental prosthetics, Diagnosis of non‐dental origin and unknown diagnosis (all at 13%)
TMD/TMJ conditions	Irreversible pulpitis, pulpal necrosis and apical periodontitis (33%)
Postoperative complications including dry socket	Irreversible pulpitis, pulpal necrosis and apical periodontitis (36%)
Mobile and exfoliating deciduous teeth	Irreversible pulpitis, pulpal necrosis and apical periodontitis (15%)
Orthodontic concerns including orthodontic appliances	Nil

## DISCUSSION

Since the beginning of the COVID‐19 pandemic, the use of telehealth in dentistry has become increasingly prevalent, allowing dental professionals to remotely screen and consult with patients. Within the context of this study, teledentistry allowed screening during the COVID‐19 pandemic, to determine the severity of presentations and to determine the need for urgent management for most patients. Recent research conducted in Melbourne, Australia, has shed light on the potential of teledentistry to support patient‐centred care and facilitate collaboration between patients, families and clinicians in designing and managing remote care plans. They found teledentistry enabled patients to initiate access to care and consult with the dental workforce remotely and safely during the peak pandemic.^27^ Prior to this current study, the researchers conducted focus groups that found teledentistry to be acceptable and feasible among public dental practitioners (blinded/redacted). Although there is some literature surrounding the validity of teledentistry globally, there are virtually no studies within Australia that have evaluated the reliability of teledentistry programs. Therefore, the findings from this study provide valuable insights into the diagnostic accuracy of teledentistry in a real‐world clinical setting.

The scope of teledentistry is vast, with extensive studies encompassing its use in various dental fields such as orthodontics, paediatric dentistry, traumatic dental injuries and oral diagnosis. Numerous studies also highlight its utility across different contexts and populations for broadening access to dental care in underserved areas. For instance, teledentistry has been utilized as a screening tool for dental caries in rural India,^28^ as well as to assess the efficacy of remote diagnosis for traumatic dental injuries using digital photographs from mobile phones,^29^ showcasing how teledentistry can facilitate timely and accurate assessments of dental trauma. Additionally, significant cost savings have also been reported in school dental screenings through a teledentistry model in Australia,^3^ underscoring its economic benefits and potential to enhance the efficiency of dental healthcare delivery. The literature also illustrates the adaptability and effectiveness of teledentistry in diverse settings, such as during oral and maxillofacial surgery consultations,^30^ the provision of interceptive orthodontic services to disadvantaged children^31^ and the accuracy of identifying dental pathologies in residential aged care patients (Alabdullah and Jampani^10^, ^16^). A systematic review by Alabdullah and Daniel^16^ has also confirmed the validity of teledentistry, reinforcing its credibility and applicability in various dental practices.

In this study, sensitivity and specificity were applied as key measures for the diagnostic accuracy of teledentistry compared to the gold standard of face‐to‐face clinical diagnoses. The analyses within this study found teledentistry specificity to range from 74.1% to 99.9%, indicating teledentistrys' ability to correctly identify true negative cases. However, the sensitivity range was from 47.6% to 100%. Both sensitivity and specificity varied considerably across different diagnostic categories. The study also showed that the sensitivity for teledentistry to identify a diagnosis of non‐dental origin or unknown conditions exhibited much lower sensitivity at 18.8%, suggesting challenges in accurately diagnosing certain complex or obscure conditions via teledentistry.

Studies examining teledentistry's diagnostic performance have reported a wide range of sensitivity and specificity values, influenced by factors such as study design, population demographics and the specific teledentistry platforms and protocols utilized.^34^ Some studies have reported sensitivity and specificity values comparable to our findings, while others have reported higher or lower values. For example, a Turkish study by Avcı and Kaptan^35^ comparing intra‐oral examination and screening findings of face‐to‐face and mobile application‐based teledentistry found high specificity (ranging from 90% to 100%) and variable sensitivity (ranging from 70% to 95%) across different diagnostic categories. These findings align with the specificity values observed in our study, indicating consistent diagnostic accuracy in ruling out true negative cases. When comparing the results by photographs obtained for diagnosis, area under the ROC curve for pericoronitis and wisdom teeth conditions were significantly higher for those with photographs compared to those without photographs. However, none of the other conditions yielded any significant results in relation to photographs.

The absence of clinical tests and dental radiographs can affect the diagnosis reached though teledentistry, which can therefore reduce the reliability of the diagnosis of several dental conditions and pathologies. In teledentistry, pulpitis is often misdiagnosed due to the need for endodontic pain assessment, radiographic assessment and thermal/mechanical testing, or potentially due to patients already having taken analgesics to control the pain, hence masking the ‘real’ endodontic diagnosis. Similarly, periodontal disease including gingivitis and periodontitis, in the absence of obvious teeth mobility, may be misdiagnosed in teledentistry since this diagnosis is heavily reliant on conducting clinical examination and periodontal probing which establishes periodontal pocket depths and attachment loss, gingival bleeding and gingival recession, which cannot be accessed by the telephone.^32^ This has been demonstrated in our study with a lower teledentistry sensitivity value of diagnosing reversible pulpitis and hypersensitivity (58.8%) and periodontal conditions (52.2%).

Certain studies indicate that intraoral evaluations conducted via telehealth examinations are just as reliable as in‐person clinical evaluations. A retrospective review completed in 2022,^32^ found dental abscess, dry socket, tooth luxation/avulsion, tempro‐mandibular dysfunction and salivary gland disease to be the least misdiagnosed conditions, while the most frequent disagreements were related to the teledentistry diagnosis of pulpitis and periodontal disease. A literature review completed in Saudi Arabia in 2021, revealed that teledentistry for dental caries detection has good sensitivity and specificity when compared to traditional visual dental examination,^33^ suggesting that teledentistry has the potential to identify some cases where immediate intervention is needed. Health service providers can use this information to tailor treatment plans and prioritize follow‐up care, ensuring timely and appropriate management of dental conditions. Nevertheless, our study did not demonstrate this higher range of diagnostic sensitivity for teledentistry, hence, it is important to consider realistic expectations in relation to its current ability to accurately diagnose the full spectrum of dental conditions.

On the other hand, an important positive finding from our study was the high specificity of teledentistry which demonstrates its potential as a valuable tool for efficiently allocating resources by accurately identifying cases that require immediate or urgent attention. For example, teledentistry can help prioritize patients with acute dental needs, ensuring that limited resources are directed towards those who need them most urgently. By accurately identifying true negative cases, teledentistry reduces unnecessary referrals and appointments, optimizing resource utilization and improving overall efficiency within the health service.

Teledentistry has shown promise for the health service to facilitate remote consultations and reduce direct contact during the COVID‐19 pandemic; however, further research is required to establish its efficacy and reliability. The findings of this study have important implications for the utilization and future application of teledentistry in dental care delivery. Our study highlights both the strengths and limitations of teledentistry as a diagnostic tool. Whilst the study presents inconclusive evidence regarding the sensitivity of using teledentistry as a mainstream method for providing broad dental diagnoses, it also demonstrates that teledentistry can serve as an excellent tool for prioritizing and triaging patients, based on the level of acuity and clinical priority. By providing a virtual platform for initial consultations, patients can receive timely advice and triaging without the barriers posed by geographical distance or mobility issues. This aspect of teledentistry holds significant potential, particularly in healthcare systems with limited resources, where prioritizing patients based on clinical urgency is essential for delivering timely and efficient care. This can streamline patient flow, reduce waiting times and optimize the use of dental resources. Teledentistry can also facilitate ongoing monitoring of patients with chronic dental conditions, which may improve patient outcomes and enhance the overall effectiveness of dental care, particularly for those requiring long‐term management of conditions, such as periodontal disease. Furthermore, virtual consultations can be used to provide tailored advice on oral hygiene, dietary choices and preventative strategies, fostering greater patient engagement.

Recommendations for future studies include the implementation of dedicated platforms for conducting teledentistry, enhanced training for dental professionals and better utilization of video and photographic media to improve diagnostic accuracy. Future studies could assess the effectiveness of initial teledentistry diagnoses in terms of treatment success, patient satisfaction and health outcomes over time. Understanding whether early teledentistry interventions lead to improved long‐term oral health can help validate its use in routine care. Additionally, future studies should seek to validate the accuracy of self‐reported symptoms during teledentistry consultations, as understanding the degree of reliability in self‐reporting will strengthen the credibility of teledentistry as a diagnostic tool. Research focusing on the efficacy of teledentistry in diverse demographic populations can inform tailored approaches, as well as conducting cost‐effectiveness studies when compared to traditional care models may provide valuable insights into its economic viability.

### Limitations

The strengths of our study lie in its large patient cohort and comprehensive analysis of teledentistry diagnostic accuracy across various demographic and diagnostic categories. However, certain limitations must be acknowledged due to the retrospective nature of the study. The study was only able to compare the teledentistry provisional diagnosis with the final diagnosis from the clinical, face‐to‐face consultation for those patients deemed to have an urgent or acute presentation during the tele‐dentistry. Patients who were deemed to have less urgent or non‐acute dental conditions during the teledentistry consultations were not offered a follow up a face‐to‐face appointment, therefore the provisional diagnosis achieved during the teledentistry couldn't be compared to a gold standard clinical examination. As a result, selection bias may arise from the retrospective nature of the study, which may lead to an over‐representation of cases where teledentistry is likely to be effective. As a result, these findings may not be generalized for the broader population of patients with chronic or less urgent dental concerns.

The study also has potential bias due to follow‐up loss, as some patients did not present to their follow up appointments. It is important to note that this finding may be influenced by potential misreporting of symptoms by participants seeking to expedite their care. Patients may exaggerate or underreport symptoms during their teledentistry consultations to influence their access to care, leading to potential inaccuracies in the data.

Dental clinicians also often record simplified notes that can be difficult to interpret or do not list all the findings within. Inconsistent documentation standards may affect the assessment of the teledentistry diagnosis in relation to the gold standard of a face‐to‐face examination. The retrospective nature of the study limited the ability to establish causality, as data was collected from existing records, which may not have captured all relevant variables.

## CONCLUSIONS

In this study, sensitivity and specificity were used as key measures for the diagnostic accuracy of teledentistry compared to face‐to‐face, clinical diagnoses. The high specificity results successfully addressed the public dental service objectives during the peak periods of the COVID‐19 pandemic, which was to identify and exclude patients with non‐urgent dental conditions from presenting to the dental clinics in person. The findings from this study may assist in the development of the current model of care utilized for acute care and emergency patients in the public sector, especially in situations with limited access to face‐to‐face dental consultations. Teledentistry may be a model of care that can be further refined and implemented into routine models, allowing for patients to be assessed, to obtain information and advice for some of their concerns remotely, as well as ensure that emergency dental appointments can be effectively utilized. The data from this study should allow further research to develop teledentistry, ultimately contributing to better oral health outcomes across diverse populations.

## AUTHOR CONTRIBUTIONS

**T Mahony:** Conceptualization; writing – original draft; writing – review and editing; methodology; project administration. **A George:** Writing – review and editing; formal analysis; supervision. **SG Kezhekkekara:** Formal analysis; software; writing – original draft. **A Villarosa:** Formal analysis; software. **C Friel:** Project administration; conceptualization. **A Yaacoub:** Conceptualization; methodology; formal analysis; supervision; writing – original draft; writing – review and editing.

## FUNDING INFORMATION

The study was funded by the Nepean Blue Mountains Local Health District.

## CONFLICT OF INTEREST STATEMENT

The authors declare that there is no conflict of interest regarding the publication of this paper.

## ETHICS STATEMENT

This study is part of a larger study which received ethics approval from the Human Research Ethics Committee of Nepean Blue Mountains Local Health District (2021/ETH00394).

## Data Availability

The quantitative data used to support the findings of this study are available from the corresponding author upon request.
